# Individual Characteristics vs. Experience: An Experimental Study on Cooperation in Prisoner's Dilemma

**DOI:** 10.3389/fpsyg.2017.00596

**Published:** 2017-04-20

**Authors:** Iván Barreda-Tarrazona, Ainhoa Jaramillo-Gutiérrez, Marina Pavan, Gerardo Sabater-Grande

**Affiliations:** ^1^LEE and Economics Department, Universitat Jaume ICastellón, Spain; ^2^Departments of Management and Economics, Center for Experimental Research in Management and Economics (CERME), Università Ca'FoscariVenezia, Italy

**Keywords:** altruism, cognitive ability, cooperation, prisoner's dilemma, experiment

## Abstract

Cooperative behavior is often assumed to depend on individuals' characteristics, such as altruism and reasoning ability. Evidence is mixed about what the precise impact of these characteristics is, as the subjects of study are generally randomly paired, generating a heterogeneous mix of the two characteristics. In this study we ex-ante create four different groups of subjects by factoring their higher or lower than the median scores in both altruism and reasoning ability. Then we use these groups in order to analyze the joint effect of the two characteristics on the individual choice of cooperating and on successful paired cooperation. Subjects belonging to each group play first 10 one-shot prisoner's dilemma (PD) games with ten random partners and then three consecutive 10-round repeated PD games with three random partners. In all games, we elicit players' beliefs regarding cooperation using an incentive compatible method. Individuals with high altruism are more optimistic about the cooperative behavior of the other player in the one-shot game. They also show higher individual cooperation and paired cooperation rates in the first repetitions of this game. Contrary to the one-shot PD games where high reasoning ability reduces the probability of playing cooperatively, the sign of the relationship is inverted in the first repeated PD game, showing that high reasoning ability individuals better adjust their behavior to the characteristics of the game they are playing. In this sense, the joint effect of reasoning ability and altruism is not linear, with reasoning ability counteracting the cooperative effect of altruism in the one-shot game and reinforcing it in the first repeated game. However, experience playing the repeated PD games takes over the two individual characteristics in explaining individual and paired cooperation. Thus, in a (PD) setting, altruism and reasoning ability significantly affect behavior in single encounters, while in repeated interactions individual and paired cooperation reach similarly high levels independently of these individual characteristics.

## Introduction

Under the assumption of common knowledge of rationality and perfect information, the only Nash equilibrium of the finitely repeated Prisoner's Dilemma (PD) is mutual defection at each stage of the game. Reasoning by backward induction, a rational player's dominant strategy is to defect at the final stage, as is also the case in the one-shot game. Knowing this, each player should also defect at the second to last round, and so on, back to the first stage.

However, some cooperative play is observed, particularly at the earliest stages, in numerous experimental tests with this game (Andreoni and Miller, 1993; Cooper et al., 1996; Pothos et al., 2011, among others). One way to reconcile the theory with the experimental evidence is to assume some kind of incomplete information. If one player does not know the true payoffs of the opponent, for example, and assigns a positive probability that the other will not defect, mutual cooperation can be sustained as equilibrium (Kreps et al., 1982). One possible interpretation of the cooperation observed in experimental games, then, is that some players are “altruistic,” in the sense that their true payoffs from cooperation are greater than the given monetary ones, and players' types are not common knowledge. Cooperation thus would be played by altruists. In a repeated game, an alternative explanation is that some players may try to “build a reputation” of cooperation in order to achieve a higher total payoff in the game. Both Andreoni and Miller (1993) and Cooper et al. (1996) use evidence of cooperation in the one-shot PD game as an indicator that a positive proportion of individuals are actually altruistic^1^. They also find that cooperation is higher when the PD is repeated for a finite number of times, consistent with reputation building. Players then turn to defection toward the end of the game, even if at a slower pace than predicted by Kreps et al. (1982). In their work, altruism is only a hypothesis to explain cooperative behavior, given that no independent measure is used to classify subjects as altruistic. However, even if altruists are expected to cooperate more, cooperation and altruism are not the same thing. Following Dreber et al. (2014) and Capraro and Marcelletti (2014), in our experiment we use as a treatment variable an ex-ante measure of altruism: giving in a Dictator Game. That is, we define as altruism the willingness to sacrifice one's own payoff in order to increase the other's payoff. Furthermore, we elicit subjects' beliefs in the PD games in order to better understand the relationship between altruism and cooperative behavior.

Several laboratory experiments have been conducted to analyze whether the cooperators in a repeated Prisoner's Dilemma (RPD) can be identified by some kind of measurable characteristics. Dreber et al. (2014) find that altruism leads to more cooperation in a noisy version of the infinitely repeated PD game only if no cooperative equilibrium exists. However, altruism does not play any role in determining the outcome when cooperation can be sustained in equilibrium. Their results support the view that social preferences are not important predictors of cooperation. Rather, individuals seem to cooperate mainly driven by payoff maximization motives. Using a dictator game to measure altruism and a standard PD game to measure cooperation, Capraro and Marcelletti (2014) find that being recipient of an altruistic act does not increase your probability of being cooperative with a third party.

We analyze the effect of altruism on cooperation, defined as the willingness to increase the joint payoffs of yourself and the other, which can be observed using one-shot and finitely repeated PD games. According to the Social Value Orientation (SVO) literature, prosocial individuals tend to maximize outcomes for both themselves and others (Van Lange et al., 1997). Eliciting beliefs about partner's cooperation allows us to tell apart whether participants classified as altruists in our study cooperate conditionally, i.e., based on the expectation that the other will also cooperate, or unconditionally, that is, even if thinking that the other will defect^2^.

Individuals' cognitive ability/intelligence has also been associated with cooperative play. One natural supposition is that more intelligent individuals should make more “rational” choices, exhibiting behavior consistent with game theoretic predictions, such as the sub-game perfect Nash equilibrium (Burks et al., 2009; Proto et al., 2015). Accordingly, these individuals should be observed to cooperate less in both one-shot and finitely repeated PD games. The empirical evidence, however, does not seem to support this conjecture. For instance, using a meta-study of repeated (PD) experiments run at numerous universities, Jones (2008, 2013) suggests that the average intelligence of game participants should be considered among the most robust factors driving individual cooperation. Specifically, this author finds that students at schools with higher average scores in the Scholastic Assessment Test (SAT) and the American College Test (ACT) tended to cooperate more often in a RPD^3^. Using a sample of 1,000 truck driving students in a one-shot sequential (PD), Burks et al. (2009) find that subjects with higher IQ more accurately forecast others' decisions and differentiate their actions more strongly given the first-mover's choice, exhibiting behavior that is far from the sub-game perfect equilibrium of the game.

Other experimental studies find mixed evidence regarding the conjecture about the negative link between cognitive ability and cooperative play. Yamagishi et al. (2014) find that decisions coherent with the maximization of self-interest are linked indeed to higher IQ. However, psychological assessment of the participants in their study leads to the conclusion that those classified as “Homo Economicus” might behave in a selfish manner only in a situation in which no future consequence of their choice is expected. These subjects can better assess the future and adopt long-term strategies. In the same line, using an experimental design similar to ours but with just one factor (participants are allocated into two groups according to their level of intelligence), Proto et al. (2015) find that higher intelligence groups do not cooperate more in the initial rounds of an infinitely repeated PD game, but seem to learn better how to reciprocate their partner's behavior over time. However, there are no significant differences in the same design with lower continuation probability. Also recently and in contrast with Jones (2008, 2013), Al-Ubaydli et al. (2016) find that cognitive ability does not predict individual cooperation in a 10-round PD game but paired cooperation is positively correlated with the average cognitive ability of the two players. In their study, individuals with higher cognitive abilities reciprocate cooperation in the second round of the PD game significantly more than low cognitive ability subjects, like in Burks et al. (2009).

Given the previous findings, an alternative conjecture is then that more intelligent individuals better adapt to the circumstances in strategic situations^4^.

Our objective in this paper is to test the significance of the joint effect of cognitive ability and altruism on cooperative behavior in a series of one-shot and finitely repeated PD games. In order to do so, both characteristics are implemented as treatment variables, separating individuals in four distinct groups based on the interaction of their high/low level of cognitive ability (measured with the Differential Aptitude Test on Abstract Reasoning), and their high/low altruistic giving in a Dictator Game (DG). In the aforementioned literature, altruism or cognitive ability or both are treated as control variables rather than treatment variables, or not taken into account. Our 2 by 2 factorial design matches individuals with similar cognitive ability and level of altruism, allowing us to neatly observe the effect of these factors on cooperation. In other words, the effect of a high reasoning ability individual with high altruism might get diluted if she found for instance a low intelligence low altruism partner when playing a RPD. Our study tries to avoid this problem.

Subjects belonging to each group played 10 one-shot PD games and three 10-round repeated PD games where we elicited players' beliefs using an incentive compatible method. Our paper is the first introducing players' beliefs to analyze expectations and behavioral rules in the RPD game under different treatments of altruism and reasoning ability.

Based on the previous review, in our study we propose the following hypotheses:

**Hypothesis 1:** *High altruism individuals should cooperate more in both one-shot and repeated PD*.

Given our definition, an altruist should be willing to increase the other's payoff at the cost of decreasing her own expected payoff, which is exactly what happens when an individual chooses the dominated cooperative strategy in our PD games.

**Hypothesis 2:** *Individuals with higher cognitive ability should more accurately forecast their partner's actions in both types of games (one-shot and repeated), and thus be able to differentiate their behavior accordingly*.

We assume that making better predictions is a necessary pre-condition to adapt successfully to a strategic situation. In line with Proto et al. (2015), we consider that more intelligent individuals should be capable of better assessing and adapting to the environment. Thus, they should better realize the scope for reputation building in the repeated game as opposed to the one-shot game.

**Hypothesis 3:** *Reasoning ability should counteract the effect of altruism in the one-shot game, while it should reinforce it in the repeated PD game*.

Our first two hypotheses propose that, while altruism should always increase cooperation, reasoning ability should lead to increased or decreased cooperation depending on the circumstances. This implies a non-linear interaction between the two factors.

Our results confirm the two first hypotheses using a clean experimental design. Reasoning ability is found to indeed counteract the effect of altruism in the one-shot games, but to reinforce it only in the first RPD. In general, the effect of the individual characteristics on the cooperation decision fades out with the repetition of the RPD game.

The article is organized as follows: Section Methods describes the experimental design and Section Results presents the results. Section Discussion discusses the results and concludes.

## Methods

We turn to experimental economics methodology to create a controlled, saliently motivated and replicable environment in which to test our hypotheses. As a first step, we used an experimental setting to measure our subjects' reasoning ability and altruism. After creating four different groups according to the results of these measures, we invited again the same subjects to the lab for a different experiment. In this second step, subjects were randomly paired with other subjects of similar reasoning ability and altruism, without them knowing this information, and played four sets of (PD) games both one-shot and repeated. Thus, each subject whose data we present in this study has participated in two sessions in different days of two consecutive weeks in December 2014: all sessions of the second experiment were carried out during the week after the last session of the first experiment. As the participants did not receive any payment up to the end of the second session, the attrition rate was low: out of 178 subjects who participated in the first set of sessions, only 16 did not participate in the second set of sessions. Subjects were recruited among undergraduate students from different degrees at Universitat Jaume I (Spain), using ORSEE (Greiner, 2015). At the beginning of each session, subjects were given written instructions, which were also read aloud by the organizers. Any remaining questions were privately answered.

At the end of the second session, subjects found out their actual gains and were privately paid in cash the total amount obtained in both sessions. Average earnings were around 11€ for the first experiment and around 14€ for the second one, and the sessions lasted 1 and 1 h and a half, respectively. Experiments were computerized and carried out in a specialized computer lab (LEE at Universitat Jaume I), using software based on the Z-Tree toolbox by Fischbacher (2007).

Each of the two experimental designs is described in detail in the following subsections. Experimental instructions can be found in Section 1 of the Supplementary Material.

### Testing for reasoning ability and altruism

In the first experimental setting, subjects were asked to complete two tasks. The first task consisted in completing the Abstract Reasoning part of the Differential Aptitude Test for Personnel and Career Assessment (DAT-AR for PCA, Bennett et al., 1974). The Abstract Reasoning (AR) scale of the DAT used in this experiment is included in the DAT-5 Spanish adaptation by the publisher TEA (Cordero and Corral, 2006). This test is usually used as a non-verbal measure of reasoning ability and involves the capacity to think logically and to perceive relationships in abstract figure patterns. It is considered as a marker of fluid intelligence (Colom et al., 2007), the component of intelligence most related to general intelligence or g factor (McGrew, 2009). The advantage of this test is that it is quite fast to implement: it is comprised of 40 multiple-choice items and has a 20 min time limit. Subjects were informed that they would receive 0.25€ for each right answer.

The second task included a Dictator Game where each subject played both as dictator (which we more neutrally called “sender”) and recipient, and then was randomly assigned one of the two roles. An endowment of 10€ was provided to dictators, who could transfer any amount from 0 to 10€ to their respective anonymous recipient in increments of 0.1€. Subjects were informed that in this task the recipient would receive no payment other than the one they chose to give. In our analysis we use the amount given in the dictator game as a measure of subjects' altruism. The dictator game is positively correlated to altruistic acts in real-life situations (returning money to subjects in Franzen and Pointner (2013) using the misdirected letter technique), charitable giving (Benz and Meier, 2008) and willingness to help in a real-effort task (Peysakhovich et al., 2014). Additionally, Carpenter et al. (2008) find that the specific survey questions for altruism used in their study are positively correlated with DG giving. Using a related concept, Capraro et al. (2014) find benevolence to be correlated with cooperative behavior, but their definition of benevolence “to increase the benefit of someone else beyond one's own” has no cost to the “benevolent” player. We consider that a person acts altruistically if she unilaterally pays a cost c ≥ 0 to increase the benefit of someone else. More formally, Player 1 is altruist toward Player 2 if she prefers the allocation (x1-c, c) to the allocation (x1, 0), where c > 0. The larger the c, the more altruist we consider this subject to be.

After completing the aforementioned tasks, subjects were divided in four groups according to their reasoning ability and altruism and called again to the lab. Apart from 16 who decided not to continue with the second session and just came separately to the lab to get their gains in the first session, the rest continued. A subject was classified as “high altruism” if she chose to transfer more than the median transferred amount in the dictator game, and as “high reasoning” if her score was higher than the median score in the DAT-AR test. Following this classification, the final four treatment groups are named “Low Altruism and Low Reasoning” (LALR, 42 subjects), “Low Altruism and High Reasoning” (LAHR, 46 subjects), “High Altruism and Low Reasoning” (HALR, 42 subjects) and “High Altruism and High Reasoning” (HAHR, 32 subjects). Therefore, a total of 162 subjects (81 pairs of players) took part in the PD sessions. Subjects were not aware at any point of the existence of the four treatments. We could not control the gender composition of each treatment but it turned out quite balanced, always in the 60–40% of females range. In Table 1 we summarize the treatments implemented.

**Table 1:** **Treatments summary**.

**Treatment**	**Subjects**	**Female (%)**	**Altruism**	**Reasoning ability**
LALR	42	43	Low	Low
LAHR	46	40	Low	High
HALR	42	59	High	Low
HAHR	32	47	High	High

### PD games

We organized 8 PD sessions, 2 for each treatment group. Each PD session began with training questions on the PD to make sure that players fully understood the mechanism of the game. Then, subjects belonging to the same treatment group were faced with four consecutive PD tasks. Subjects were informed that they would be paid according to their decisions in only one of the four tasks, randomly selected at the end of their session.

#### One-shot PD games

The first task consisted in a sequence of 10 one-shot PD games against potentially different anonymous opponents using a strangers-pairing mechanism. No player knew the identity of the player with whom she was currently paired or the history of decisions made by any of the other players.

Table 2 shows the payoffs of the one-shot PD game. In each cell, the first (second) figure denotes the payoff in euros of player 1 (2). Clearly from the Table, “A” represents the decision to cooperate and “B” not to cooperate.

**Table 2:** **Payoffs of the one-shot game**.

**Player 1**	**Player 2**	
	**A**	**B**
A	(20, 20)	(0, 28)
B	(28, 0)	(10, 10)

In order to avoid endowment effects across the one-shot games in this task, we used the RLI (Random Lottery Incentive) system as payment mechanism. That is, if this task was selected for payment, only one randomly drawn PD game was remunerated. We didn't randomize task order and made all players play this task first, so that subjects could face a great number of opponents (up to 10 different ones) and in this way get some information about the population of players that they were facing.

#### Finitely repeated PD games

In the last three tasks participants played a repeated PD game, in which each subject played 10 rounds of the same game with a given participant using a partners-pairing mechanism. Therefore, each subject played 10 consecutive rounds with the same opponent. Players were then anonymously re-matched with new opponents and played a new RPD lasting again 10 rounds. At the end of each period in a repetition, subjects were shown what their opponent had played. However, when players were re-matched, they were not told anything about the history of play of their new opponent.

The payoffs of each round for all three RPD tasks are shown in Table 3. It can be observed that they are just equal to those of a round of the one-shot game divided by ten.

**Table 3:** **Payoffs of the RPD game**.

**Player 1**	**Player 2**	
	**A**	**B**
A	(2, 2)	(0, 2.8)
B	(2.8, 0)	(1, 1)

### Beliefs

In order to gather more detailed information on players' strategic reasoning, subjects were asked the following questions before each round of each game:

1.- “Do you think your partner will choose A or B this period?”
2.- “What percentage of players will choose to play A this period?”

With the first question we elicit the “individual” belief and with the second one the “social” belief on individual cooperation.

Subjects could earn up to two additional euros for these questions, according to their answers^5^.

## Results

Before reporting the detailed results related to cooperation behavior in the (PD) tasks, we first describe the outcomes of the reasoning ability test and of the Dictator Game, and subjects' beliefs in the PD tasks.

### Descriptive statistics

**Figure 1:**
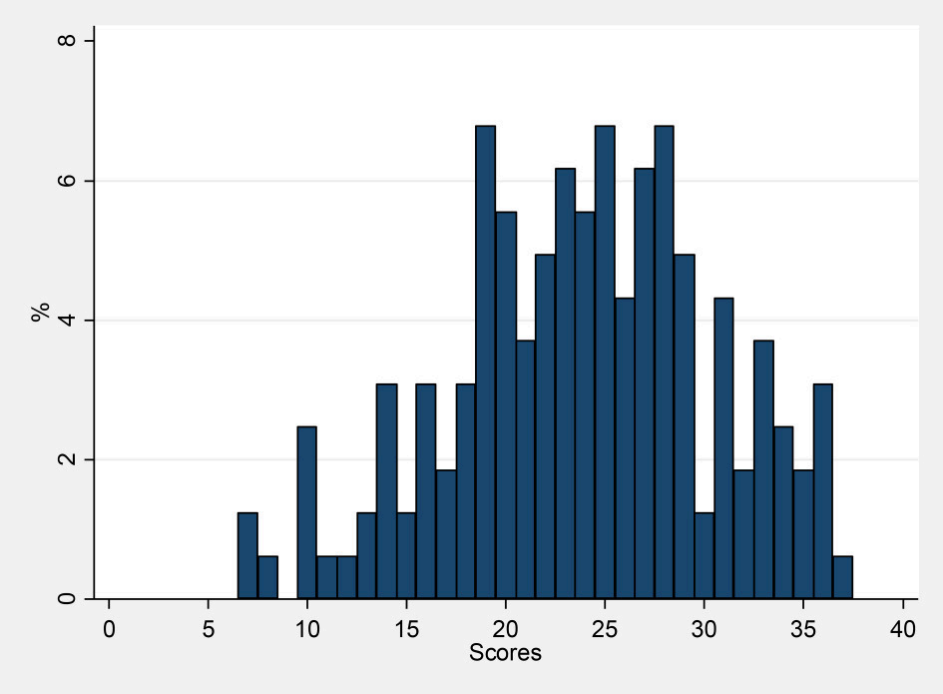
**Scores observed in the DAT-AR test**.

Figure 1 presents the distribution of the number of observed correct answers to the 40 multiple choice items in the DAT-AR test. The mean and the median number of right answers were 23.9 and 24 out of 40, respectively, and the standard deviation was 6.7. Mean and median number of correct answers are almost identical to the ones calculated for the Spanish population of a comparable age (Cordero and Corral, 2006).

**Figure 2:**
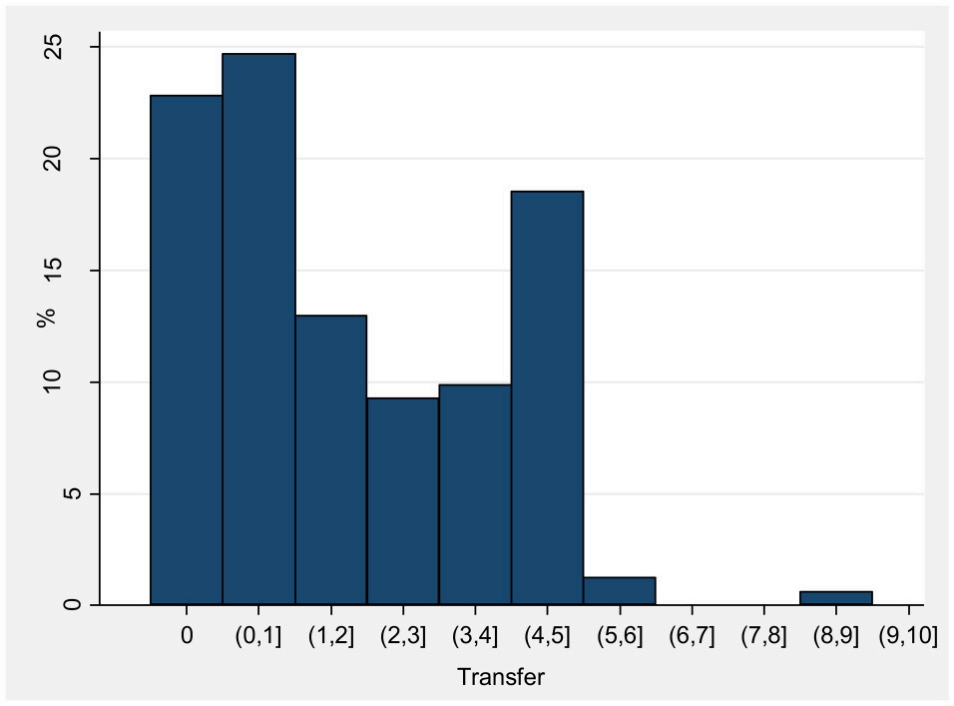
**Number of subjects per transfer interval in the Dictator Game**.

Figure 2 shows the distribution of the transfers in the Dictator Game. About 80% of our subjects gave non-zero amounts. The mean and median transfer were of 2 and 1.4€ out of 10€, respectively, and the standard deviation was almost 2€. Comparing these results with the range of outcomes in the dictator game meta-analysis of Engel (2011), our values are within the range of what is typically observed (dictators on average give 28.35% of the pie).

Table 4 shows descriptive statistics on reasoning ability and altruism for subjects included in the four treatment groups. On average, “high” altruism subjects transfer about 3€ more than “low” altruism ones, while subjects with “high” reasoning ability answered correctly to about 10 additional questions with respect to subjects with “low” reasoning ability. Comparing these results with the general ones for Spain from Cordero and Corral (2006), 19 correct answers correspond to about the 25% percentile of the DAT-AR scores distribution, and 29 correct answers to about the 75% percentile.

**Table 4:** **Altruism (A) and Reasoning ability (R) descriptive statistics by treatment**.

	**Mean**		**S.D**		**Min**		**Max**	
	**A**	**R**	**A**	**R**	**A**	**R**	**A**	**R**
LALR	0.45	18.74	0.52	4.85	0.0	7	1.5	24
LAHR	0.43	29.67	0.53	3.33	0.0	25	1.5	37
HALR	4.15	18.62	1.24	3.99	2.0	8	8.2	24
HAHR	3.52	29.09	1.19	3.77	2.0	25	6.0	36

For the pooled data, there is a significantly negative correlation between altruism and reasoning ability, but it is quite low (Spearman's rho of −0.17, *p* = 0.032). Besides, the correlation between the two characteristics is not significant within each group. However, we test for collinearity in our regression analysis.

### Beliefs

**Figure 3:**
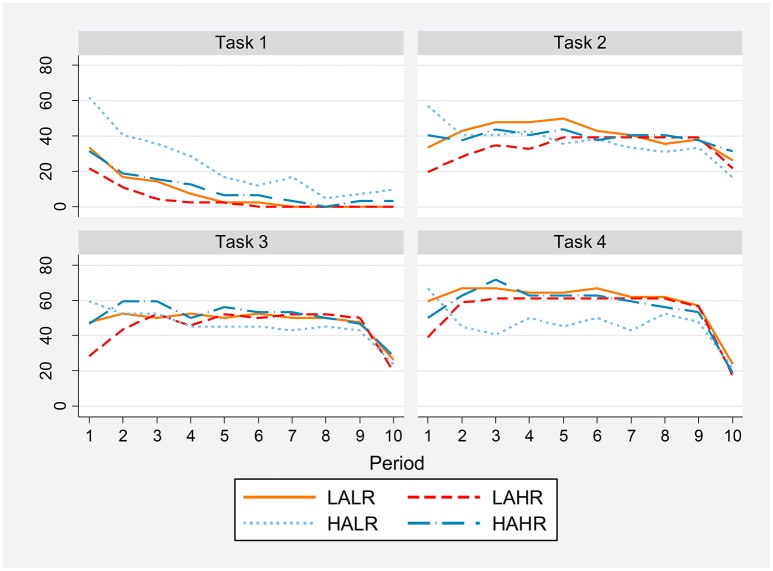
**Percentage of individuals whose belief is partner cooperation in the current period by task, period and treatment**.

Figure 3 shows the percentage of participants whose belief is that their partner will cooperate in that particular period (the “individual belief,” that is, the answer to question 1 reported in Section Beliefs above) by task, period and treatment. In the one-shot game high altruism individuals with low reasoning ability (HALR) have a higher expectation of partner cooperation than the rest. This difference is significant for the first seven periods when we compare HALR vs. LALR (with the exception of period 6) and HALR vs. LAHR using a proportion test, and for the first period when we compare HALR vs. HAHR. The full test statistics are presented in Table SM2.1 in the Supplementary Material (all our tests *p*-values have been Bonferroni corrected to take into account the problem of false positives in multiple comparisons).

In the first period of each RPD task we observe that HALR individuals continue to have the most positive expectations about partner cooperation, while LAHR subjects are the most pessimistic, this difference being significant for tasks 2, 3, and 4 (see the proportions tests results in Tables SM2.2–SM2.4 in the Supplementary Material). However, these treatment differences level off over time within each RPD game.

On average over all periods in a task, high reasoning ability subjects have a lower expectation of partner's cooperation in the one-shot game (Mann-Whitney test *z* = −4.034 and *p* = 0.0001), while there are no significant differences in expectations in the repeated PDs. This shows that HR individuals' beliefs are more consistent with the Nash equilibrium of the game, but only in the one-shot.

The mean percentage of individuals expected to cooperate in each period (the “social belief,” that is, the answer to the second question reported in Section Beliefs), shows a similar pattern to that of the individual belief (see Figure SM2.1 in the Supplementary Material).

**Figure 4:**
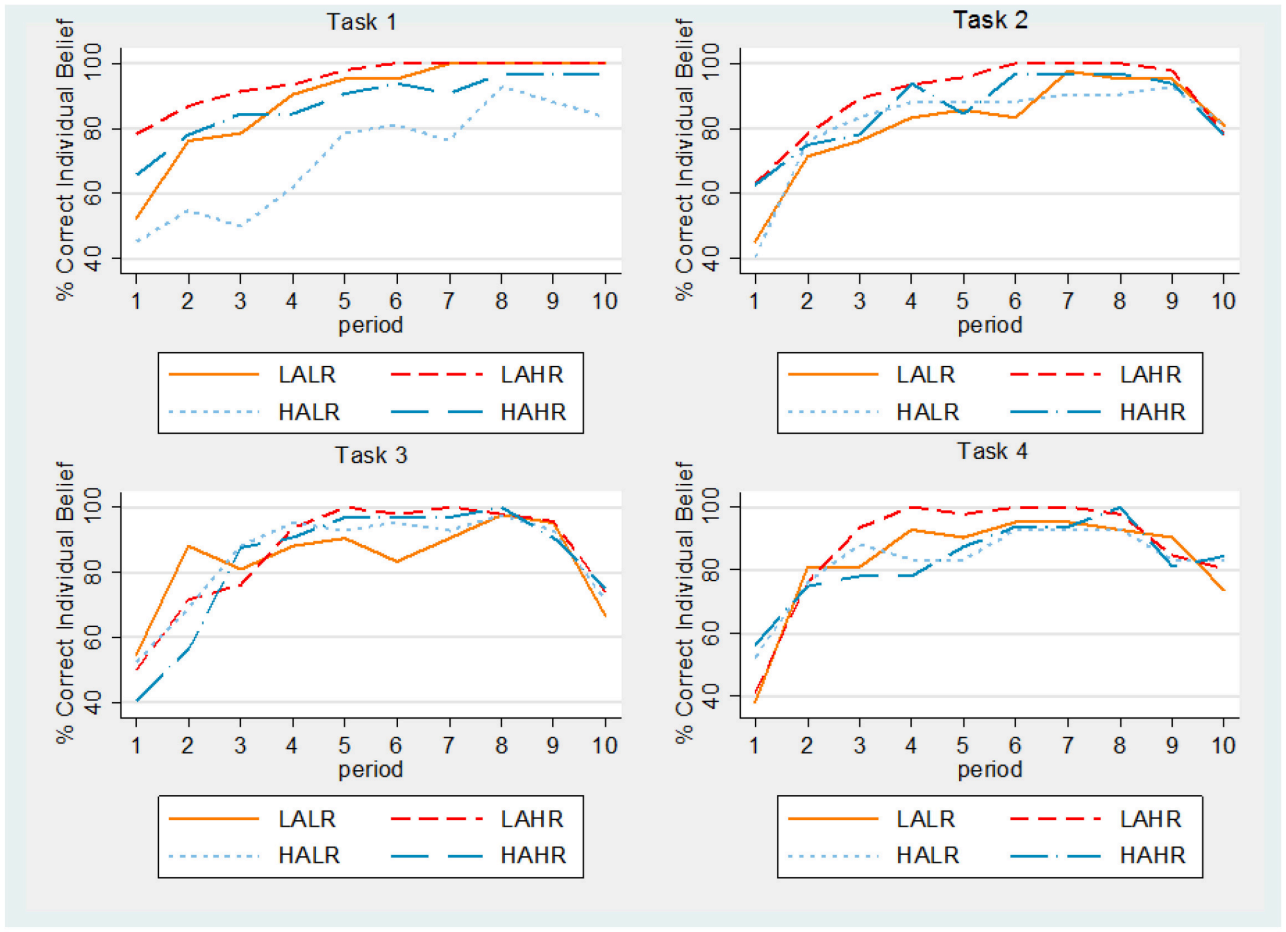
**Percentage of correct individual beliefs by task, period and treatment**.

The elicitation of beliefs allows us to measure the number of individuals who have correctly guessed their partner's behavior in any given period, that is, they expected cooperation and the other has indeed cooperated, or they expected defection and the other has defected. Dividing this number by the total number of individuals in the treatment, we obtain the percentage of correct beliefs for each task, period and treatment (presented in Figure 4). According to Hypothesis 2 in the Introduction, we should observe that individuals with higher cognitive ability better forecast their partner's behavior. The percentage of correct individual beliefs is significantly higher for high reasoning ability subjects in the first four repetitions of the one-shot game (see Table SM2.5 in the Supplementary Material) and in the first period of task 2. In particular, LAHR participants reach 100% accuracy in almost half of the periods in all tasks, more often than the other treatments. However, there are no systematic differences in the remaining periods and tasks (Tables SM2.6–SM2.8 in the Supplementary Material). In the RPD tasks, the percentage of correct guesses is above 80% for most periods, for all treatments.

**Result 1:** *High cognitive ability subjects better forecast their partner's behavior in the first repetitions of the one-shot games and at the beginning of the first RPD. However, there are no systematic differences in the percentages of correct guesses in the remaining repetitions of the RPD*.

Notice that high altruism individuals with low reasoning ability less accurately forecast their partner's behavior in task 1. This is consistent with the fact that they have a too optimistic view of their partner's behavior in the one-shot game.

### Individual cooperation in period 1 of each task

**Figure 5:**
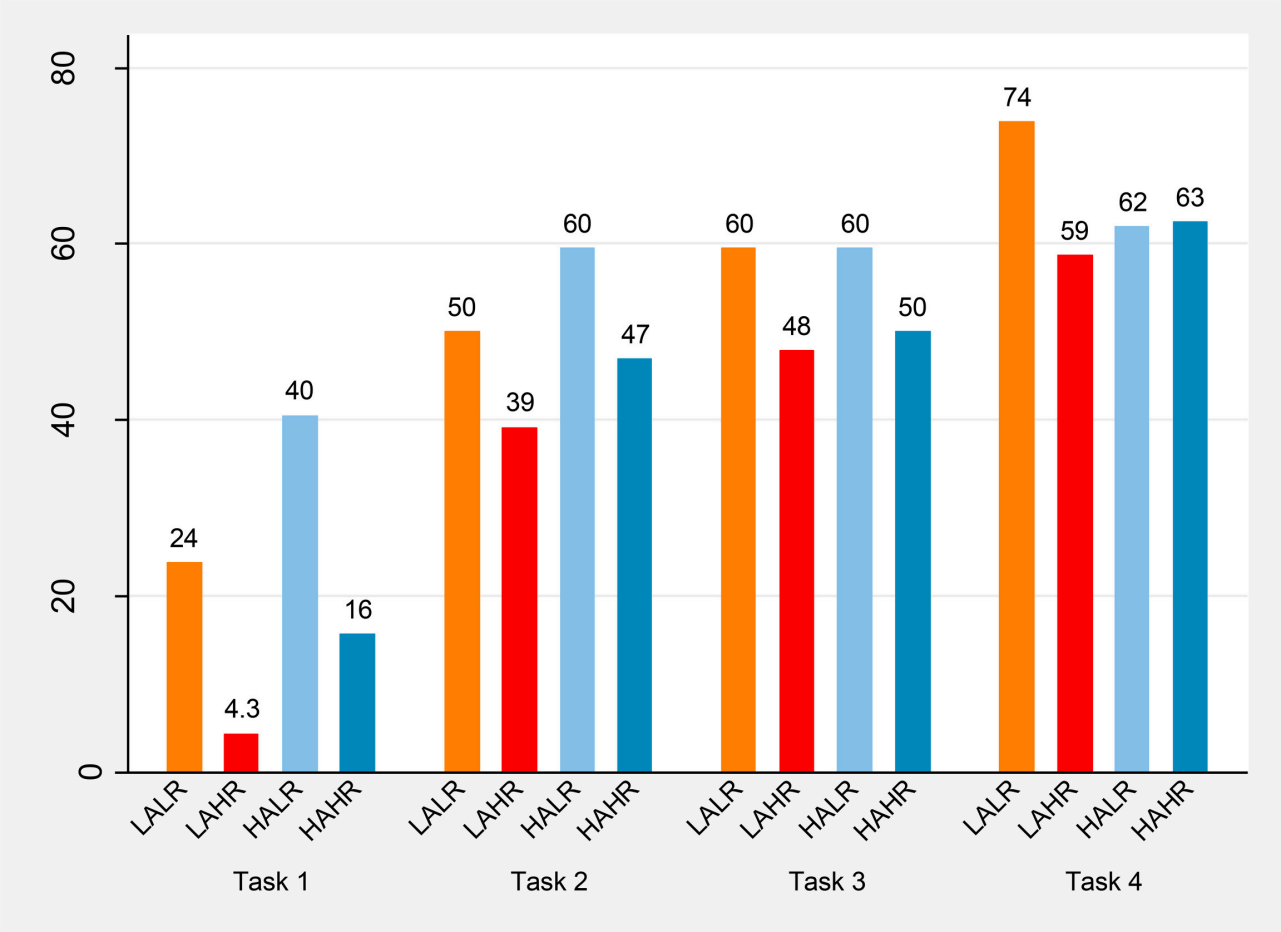
**Percentage of individuals cooperating in period 1 of each task**.

In Figure 5 we present the percentage of subjects choosing to cooperate in period 1 for each task and treatment.

The observed level of cooperation in the very first one-shot PD game depends on both altruism and reasoning ability.

**Result 2:** *In the first PD game altruism tends to increase cooperation while reasoning ability tends to decrease it*.

Coherently with our Hypotheses 1 and 3, in the first one-shot PD game high altruism subjects cooperate more than low altruism subjects, and high reasoning ability subjects cooperate less than low reasoning ability ones. These differences are significant using a proportion test, as reported in SM2.12 (period 1).

**Result 3:** *Individual cooperation rates are higher at the beginning of RPD games than at the beginning of the sequence of one-shot PD games, particularly for high reasoning ability subjects*.

Using a proportion test we obtain that the percentage of individuals cooperating in period 1 is significantly higher in all repeated PD tasks than in task 1 for all treatments with the exception of the HALR treatment (see Table SM2.9 in the Supplementary Material). After a significant increase in first period cooperation from task 1 to task 2 especially for high reasoning ability subjects, the cooperation level remains stable at the beginning of the remaining tasks. Consistently with our Hypothesis 2, we observe a more marked difference in behavior between the one-shot and the repeated tasks for high reasoning ability individuals.

The observed differences in cooperation for the first one-shot PD game are no longer significant for the first period of each repeated game. The high reasoning ability subjects, who cooperated significantly less at the beginning of the one-shot games, show no significantly lower cooperation levels at the beginning of the subsequent tasks (tests results are available upon request). High reasoning ability individuals seem to better anticipate the lower cooperation rate that will be attained in a series of one-shot games with different partners as opposed to a sequence of repeated interactions with the same partner.

### Individual cooperation dynamics

**Figure 6:**
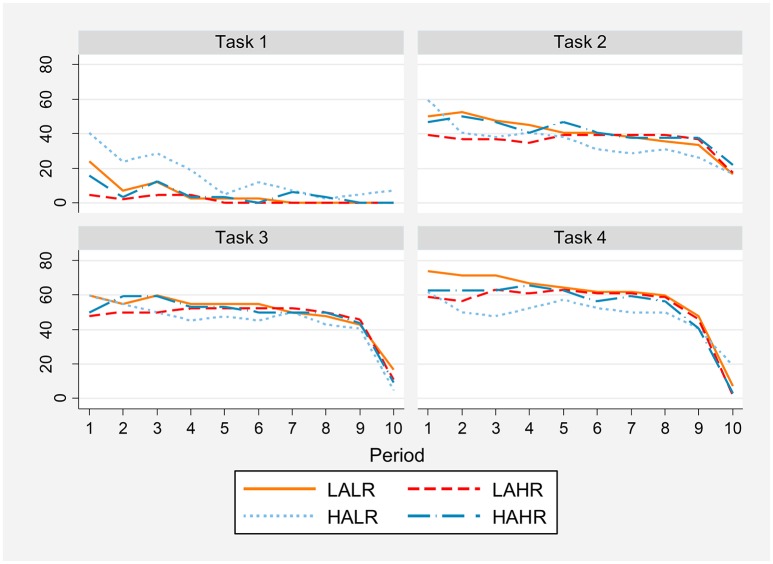
**Percentage of individual cooperation by task, period and treatment**.

Figure 6 shows individual cooperation percentages by task, period and treatment.

The percentage of cooperation decreases for all treatments as the one-shot PD game is repeated (task 1). However, the group with higher altruism and lower reasoning ability never reaches a 0% individual cooperation rate (the other treatment groups reach 0% individual cooperation in periods 5 to 9). Table SM2.10 in the Supplementary Material shows percentages of individual cooperation in the repetitions of the one-shot game, for all treatments.

Using a proportion test, in Table SM2.12 in the Supplementary Material we show that high reasoning ability participants (HR) cooperate significantly less in the one-shot PD game than low reasoning ability ones (LR) in the first two repetitions (column 1). Additionally, the percentage of cooperation is significantly higher for high altruism subjects (HA) than for low altruism ones (LA) for several periods, as can be seen in column 4.

As can be observed in Figure 6, in the RPD tasks individual cooperation not only is higher at the beginning but also sustained at around 40% to 60% until the very last period, when it falls abruptly (see details in Table SM2.11 in the Supplementary Material). However, last period individual cooperation rates are still positive, differently from task 1, for most treatments. No significant treatment effects appear in the RPD tasks, as we had already observed in our analysis of period one.

#### Regression analysis

In order to account for the effect of beliefs and of the stage game repetitions within each task together with the treatment, we run random-effects panel logit regressions. Results are reported in Table 5.

**Table 5:** **Random-effects panel logit regressions of individual cooperation on treatment, period and beliefs**.

**Individual cooperation**	**Task 1**		**Task 2**		**Task 3**		**Task 4**	
	**Coeff**.	**Sd.E**.	**Coeff**.	**Sd.E**.	**Coeff**.	**Sd.E**.	**Coeff**.	**Sd.E**.
Social belief	0.02^***^	(0.01)						
Individual belief			5.31^***^	(0.31)	5.15^***^	(0.28)	5.19^***^	(0.29)
Female	0.23	(0.34)	0.18	(0.36)	0.01	(0.33)	−0.38	(0.33)
Period	−0.41^***^	(0.05)	−0.32^***^	(0.04)	−0.31^***^	(0.04)	−0.37^***^	(0.04)
LAHR	−1.52^**^	(0.60)	0.15	(0.49)	−0.23	(0.43)	−0.39	(0.44)
HALR	1.13^***^	(0.43)	−0.33	(0.50)	−0.52	(0.45)	−0.30	(0.46)
HAHR	−0.24	(0.52)	0.28	(0.53)	−0.64	(0.48)	−0.60	(0.49)
Constant	−2.62^***^	(0.46)	−1.64^***^	(0.42)	−0.84^**^	(0.39)	−0.17	(0.41)
N	1620		1620		1620		1620	
Wald Chi^2^	96.72^***^		295.07^***^		341.80^***^		320.63^***^	

*** *Coefficient significant at 1%*,

** *Significant at 5%. Standard errors in parentheses*.

The variables used are the following:

- Individual cooperation: dependent variable. Takes value 1 when the individual decides to cooperate in the current period, 0 otherwise.
- Social belief: individual expectation on the percentage of subjects cooperating in the current period and session. Ranges from 0 to 100.
- Individual belief: takes value 1 if the individual expects the partner to cooperate in the current period, 0 otherwise.
- Female: takes value 1 if the subject is female, 0 otherwise.
- Period: takes values 1 to 10 in each task.
- LAHR/HALR/HAHR: dummy variables that take value 1 for the corresponding treatment, 0 otherwise.

In the regression for task 1 (the one-shot PD game) we consider “social belief” more appropriate than “individual belief” as a regressor, given that the individual is not always playing with a same partner.

The baseline treatment is “Low Altruism and Low Reasoning” (LALR). Within the “Low Altruism” subjects, the treatment with “High Reasoning” (LAHR) shows significantly lower cooperation in the one-shot PD game. On the opposite, a high level of altruism significantly increases the probability of cooperating for individuals characterized by “Low Reasoning” ability (HALR vs. the baseline LALR). The joint effect of high reasoning ability and high altruism appears to be null. In fact, there are no significant differences in cooperation between HAHR and LALR subjects, which could be due to the fact that the effects of a higher reasoning ability and a higher altruism go in opposite directions. This is coherent with the interaction effect we anticipated in Hypothesis 3.

We also observe that the higher the expectation on the percentage of players cooperating in that round, the higher individual cooperation. Moreover, each additional period significantly reduces the likelihood of cooperation. Gender has no significant effect.

Treatment effects disappear in the RPD tasks: none of the estimated coefficients for each of the three treatment dummies is significantly different from zero. In these tasks, thinking that the partner will cooperate significantly rises the probability of cooperation. There is a negative significant effect of period.

We can directly include reasoning ability and altruism measurements in these regressions rather than using a dummy for each group. Results are reported in Table 6. The variables used to measure reasoning ability and altruism are the following:

- Reasoning ability: number of correct answers in the DAT-RA test. Ranges from 7 to 37.
- Altruism: euros transferred to the recipient in the dictator game. Ranges from 0 to 8.2.

**Table 6:** **Random-effects panel logit regressions of individual cooperation on individual characteristics, period and beliefs**.

**Individual cooperation**	**Task 1**		**Task 2**		**Task 3**		**Task 4**	
	**Coeff.**	**Sd.E.**	**Coeff.**	**Sd.E.**	**Coeff.**	**Sd.E.**	**Coeff.**	**Sd.E.**
Reasoning ability	−0.09^***^	(0.03)	0.06^**^	(0.03)	0.04	(0.02)	−0.00	(0.02)
Altruism	0.20^**^	(0.09)	0.00	(0.09)	−0.01	(0.08)	−0.04	(0.09)
Social belief	0.02^***^	(0.01)						
Individual belief			5.31^***^	(0.31)	5.11^***^	(0.28)	5.17^***^	(0.29)
Female	0.31	(0.36)	0.20	(0.36)	0.02	(0.33)	−0.36	(0.33)
Period	−0.41^***^	(0.05)	−0.32^***^	(0.04)	−0.31^***^	(0.04)	−0.37^***^	(0.04)
Constant	−1.13	(0.80)	−3.03^***^	(0.76)	−2.00^***^	(0.70)	−0.36	(0.71)
N	1620		1620		1620		1620	
Wald Chi^2^	91.06^***^		297.67^***^		342.84^***^		319.96^***^	

*** *Coefficient significant at 1%*,

** *Significant at 5%. Standard errors in parentheses*.

Although the correlation between reasoning ability and altruism was weak, we tested for collinearity in the estimated models. Results of these tests are reported in Table SM2.13 in the Supplementary Material. The Variance Inflation Factors are quite low (slightly above 1) for all regressors, indicating that there is no cause for concern.

For task 1 we obtain that reasoning ability has a significant negative effect while altruism increases the likelihood of cooperating, thus extending our Result 2 beyond the first period to all the one-shot PD games. The effect of the remaining variables is robust to the replacement of the treatment dummies by cognitive ability and altruism variables.

**Result 4:** *In the one-shot PD games, the effect of reasoning ability on the likelihood of cooperation is negative while that of altruism is positive. Additionally, individual beliefs and period also significantly affect the cooperation decision. Gender is not relevant*.

In task 2 reasoning ability continues to be significant for explaining cooperation. However, note that the direction of the effect is the opposite, that is, higher abstract reasoning leads to less cooperation in the one-shot PD and to more cooperation in RPD, thus confirming our Hypothesis 3. As we pointed out above, it seems that subjects with higher reasoning ability better recognize the different nature of the games played and the relatively lower opportunities of coordinating on cooperation that playing with a changing partner provides. Thus, these subjects seem to better adjust their behavior to the environment.

**Result 5:** *The effect of reasoning ability on cooperation is negative in the one-shot games but positive in the first RPD task*.

In tasks 3 and 4 neither reasoning ability nor altruism affect cooperation. Instead, the belief that the partner will cooperate significantly increases the likelihood of cooperating in all tasks. In fact, this belief turns out to be highly correlated with past partner cooperation (which we have not included in the regression for this reason: Spearman's rho of 0.76, *p* < 0.001). Again, period has a significantly negative effect and gender plays no role.

**Result 6:** *Experience with the RPD game takes over individual characteristics of the subjects in explaining their decision*.

While reasoning ability significantly predicts cooperation behavior the first time the repeated game is played (task 2), individual characteristics do not seem to play a role when participants gain experience facing the RPD a second and a third time (tasks 3 and 4).

#### Unconditional cooperation

Using the information on beliefs, we computed the percentage of individuals who cooperate “unconditionally,” that is, even if expecting defection, for each period of each task. The result is that very few individuals choose to cooperate thinking that the partner will defect. In the one-shot, on average only 1.5% of low altruism and 2.8% of high altruism participants' decisions are A/B. In the repeated tasks, on average <6% of both high and low altruism subjects' decisions are unconditionally cooperative. We interpret this result as evidence of very low unconditional cooperation. In fact, taking into account the payoff table of the game, we can observe that even a high altruism subject would find it hard to cooperate unconditionally. On average high altruism subjects were willing to sacrifice 4€ out of 10€ in the dictator game, while in the one-shot PD they should give up 10€ and get nothing if they cooperate thinking that the partner is not going to cooperate. In fact no player gave up the whole 10€ endowment in the DG.

**Result 7:** *There is scarce evidence of unconditional cooperation, even for high altruism subjects*.

### Paired cooperation

By paired cooperation we refer to the situation where both members of a pair simultaneously decide to cooperate in a given period, thus obtaining the cooperative payoff of the Prisoners' Dilemma.

**Figure 7:**
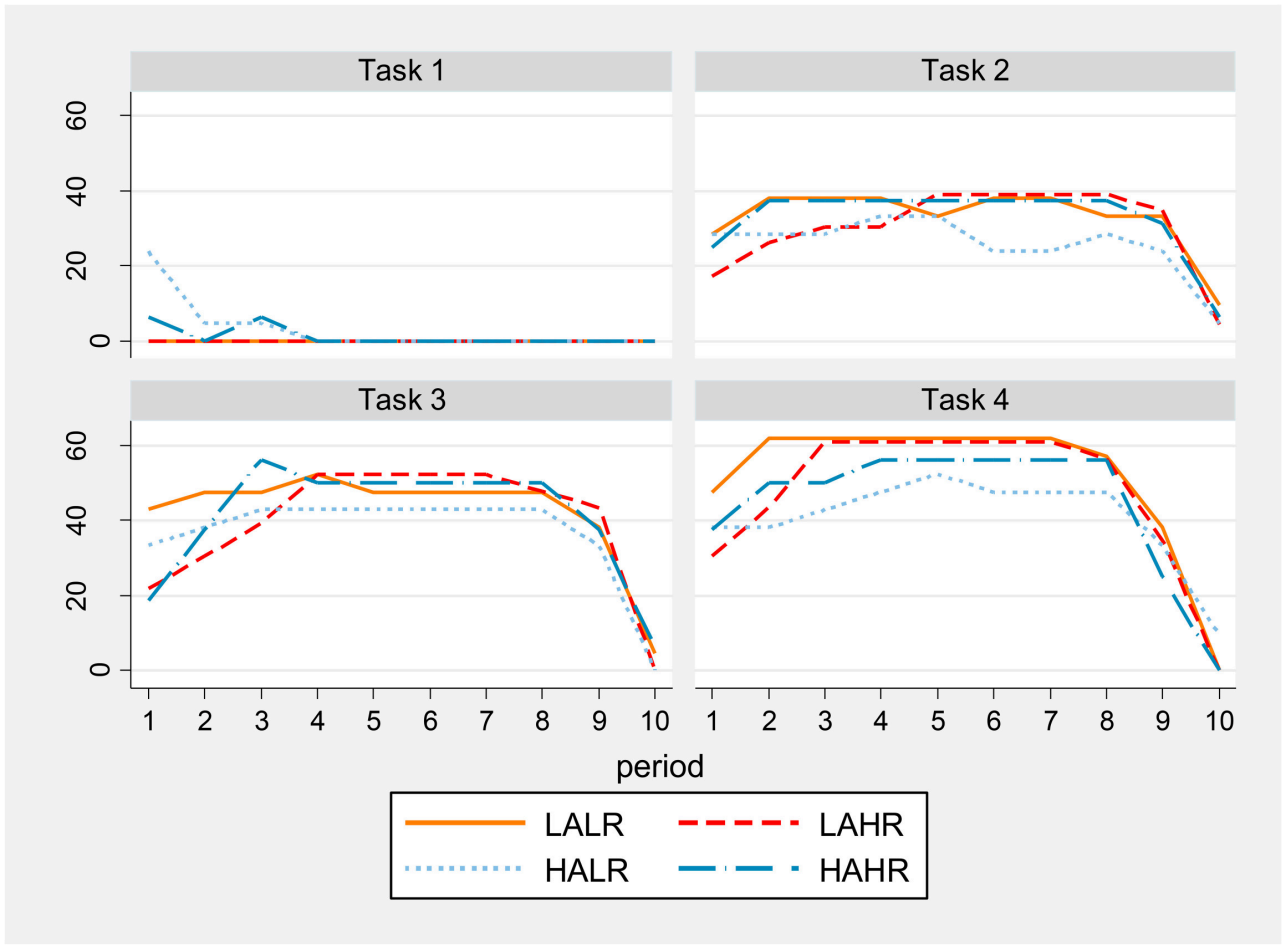
**Percentage of paired cooperation by task, period and treatment**.

As can be seen in Figure 7, successful paired cooperation is obviously much lower in the one-shot than in the repeated PD. Only altruists show some positive cooperation at the beginning of task 1. The difference in paired cooperation between low and high altruism pairs is significant for the first one-shot game (*z* = −2.78 and *p* = 0.003). All treatments increase paired cooperation at the beginning of the RPD games, particularly high reasoning ability subjects which show steep and significant increases in the first two periods. Specifically, we find significant differences comparing the level of paired cooperation in period 2 vs. period 1 for high reasoning ability pairs (at 5% in tasks 2 and 3, marginally in task 4; test details in Table SM2.14 in the Supplementary Material). There are no other treatment differences in reaching and sustaining high cooperation. Tasks 2 and 3 present levels of paired cooperation close to 40%, and task 4 reaches 60%.

**Result 8:** *In the first one-shot game high altruism subjects exhibit higher levels of paired cooperation than low altruism ones*.
**Result 9:** *In the RPD game high reasoning ability subjects significantly increase paired cooperation in the first two periods, all treatments attaining and sustaining similarly high levels until one period before the last of each repetition, when cooperation crumbles*.

## Discussion

We study cooperative behavior in (PD) games using a neat 2 by 2 factorial design, considering high vs. low altruism and high vs. low reasoning ability. As in all the previous experiments with these games, we find evidence of cooperation in both one-shot and finitely repeated (PD). In particular, we confirm the result by Andreoni and Miller (1993) and Cooper et al. (1996) that a certain amount of cooperative play appears to be due to the altruistic nature of subjects. In fact, by using an external measure of altruism (giving in a Dictator's Game), we show that altruism positively affects the likelihood of cooperation in the one-shot PD games. Moreover, high altruism players seem to be more optimistic about their partners' behavior and they cooperate mainly thinking that their partner will also cooperate. Successful paired cooperation is very low in the one-shot games, with high altruism pairs being the only ones to reach positive levels.

As in the aforementioned studies and coherent with the “reputation building” hypothesis, we find that both individual and paired cooperation rates are much higher (40–60%) in the repeated PD games, and sustained for almost all periods, only to fall sharply in the last period of each task. Thanks to the elicitation of players' beliefs, we show that in our experiment cooperation is almost never unconditional: even altruistic subjects hardly cooperate if they think that their partner is going to defect. Altruism does not significantly increase neither individual nor paired cooperation in RPDs.

Interestingly, the effect of reasoning ability on individual cooperation changes sign depending on the type of PD game. Reconciling part of the previous literature and consistently with Burks et al. (2009)'s result for sequential PD, higher cognitive ability subjects appear to better adapt to the particular game played. In particular, they more accurately forecast their partner's behavior in the first repetitions of the one-shot games and at the beginning of the first RPD. Coherently, they tend to cooperate significantly less in the one-shot PD, as hinted in the lower continuation probability treatments of Proto et al. (2015). Also, they are more likely to cooperate in the first RPD, in line with what Jones (2008) found in his analysis using average intelligence scores. Differently to Al-Ubaydli et al. (2016), where paired cooperation is predicted by cognitive ability whereas individual cooperation is not, we do not find fundamental differences between individual and paired cooperation.

Reasoning ability is found to counteract the effect of altruism in the one-shot game. In fact, the joint effect of high reasoning ability and high altruism on the likelihood of cooperation appears to be no different from that of low reasoning ability and low altruism. However, while low reasoning ability individuals display similar behavior in both one-shot and RPD games, high reasoning ability subjects appear to better understand the nature of the one-shot (PD), changing then their decisions in the repeated version of the game.

Individual characteristics, however, fast reduce their weight in affecting subjects' decisions. While both reasoning ability and altruism explain individual cooperation in the one-shot PD and reasoning ability continues to be significant in the first RPD game, both characteristics become irrelevant as explicative variables when subjects gain experience in the RPD game. Instead, the variables affecting individual cooperation are period and subject beliefs. The latter could still be mediated by subject type, but in a more dynamic and adaptive way, as beliefs in the RPD are highly correlated with past partner cooperation. With experience in the RPD, reached and sustained cooperation end up being similar among all groups. Thus, in a (PD) setting, altruism and reasoning ability significantly affect behavior in a situation in which no future consequence of choices is expected. This effect appears to be diluted when building a reputation can be used to reach higher payoffs. Indeed, transforming a social relationship into repeated interactions appears to be key to achieve mutual cooperation (Axelrod, 1984).

As future research, personality traits could also be added as determinants of cooperation, such as agreeableness or extraversion, as in Pothos et al. (2011), Proto et al. (2015), or Kagel and McGee (2014). They could be added as controls rather than as treatment variables, because the latter option would much complicate the treatment structure and impose high demands on the number of participants. An efficient alternative would be to program algorithmic players with a selection of frequently studied strategies and make them interact with human players, as in Hilbe et al. (2014). Also, having an increased age and culture variability could add insights on the determinants of cooperation.

## Ethics statements

This study was carried out in accordance with the recommendations of the ethical committee from the Universitat Jaume I. Participants gave informed consent in accordance with the Declaration of Helsinki. All participants in the subject database from the LEE at Universitat Jaume I in Castellón have voluntarily signed to participate in economic experiments and can freely decide whether they want to take part or not in each proposed experiment. No deception takes place in any experiment run at the LEE. No vulnerable populations were involved in the study.

## Author contributions

All authors collaborated in the development of the idea, the design of the project and the running of the sessions. IB programmed the software. AJ and IB developed the database and carried out most of the analyses. MP, IB, and GS wrote the article. All authors revised and accepted the written version.

## Funding

Financial support by Universitat Jaume I (project P1.1B2015-48) and the Spanish Ministry of Economics and Competitiveness (projects ECO2013-44409-P and ECO2015-68469-R) is gratefully acknowledged.

### Conflict of interest statement

The authors declare that the research was conducted in the absence of any commercial or financial relationships that could be construed as a potential conflict of interest.

## Footnotes

^1^In Cooper et al. (1996) a player is classified as an altruist (egoist) if he plays cooperatively more (less) than 50% of the time in 20 one-shot PD games. Andreoni and Miller (1993) consider any cooperation in the one-shot game as a sign of altruism.

^2^According to the SVO literature, prosocial individuals can be either altruists (unconditional cooperators) or cooperators (conditional cooperators). Differently from this literature, we do not identify altruists as unconditional cooperators.

^3^Jones (2014) increases the information processing necessary to implement strategies supporting cooperation of the RPD game through random switching between permutations of the payoff table. This additional strategic complexity attenuates the relationship between cognitive ability and cooperation observed in Jones (2008). In a related cognitive load experiment, Duffy and Smith (2014) find that a decrease in the cognitive load of subjects increases strategic defection near the end of the RPD game.

^4^Intelligence and adaptive behavior are found to be separate but related constructs exhibiting low to moderate correlations depending on the particular measures (Harrison, 1987; Keith et al., 1987; Platt et al., 1991). The underlying mechanism behind the relationship between intelligence and adaptive behavior is out of the scope of our paper.

^5^At each round of the one-shot PD, subjects received 1€ for answering the first question correctly and 1€ minus as many cents as the difference (in absolute value) between their answer to question 2 and the actual percentage of players choosing cooperation in that round. At each round of the repeated PD the stakes were one tenth of the one-shot PD, that is 0.1€ gain, and one tenth of the difference penalty.

## References

[B1] Al-Ubaydli O. Jones G. Weel J. (2016). Average player traits as predictors of cooperation in a repeated prisoner's dilemma. J. Behav. Exp. Econ. 64, 50–60. 10.1016/j.socec.2015.10.005

[B2] Andreoni J. Miller J. H. (1993). Rational cooperation in the finitely repeated prisoner's dilemma: experimental evidence. Econ. J. 103, 570–585. 10.2307/2234532

[B3] Axelrod R. (1984). The Evolution of Cooperation. New York, NY: Basic Books.

[B4] Bennett G. K. Seashore H. G. Wesman A. G. (1974). DAT: Differential Aptitude Test. New York, NY: The Psychological Corporation.

[B5] Benz M. Meier S. (2008). Do people behave in experiments as in the field? Evidence from donations. Exp. Econ. 11, 268–281. 10.1007/s10683-007-9192-y

[B6] Burks S. V. Carpenter J. P. Goette L. Rustichini A. (2009). Cognitive skills affect economic preferences, strategic behavior, and job attachment. Proc. Natl. Acad. Sci. U.S.A. 106, 7745–7750. 10.1073/pnas.0812360106 19416865 PMC2683075

[B7] Capraro V. Marcelletti A. (2014). Do good actions inspire good actions in others? Sci. Rep. 4:7470. 10.1038/srep07470 25502617 PMC4264010

[B8] Capraro V. Smyth C. Mylona K. Niblo G. A. (2014). Benevolent characteristics promote cooperative behavior among humans. PLoS ONE 9:e102881 10.1371/journal.pone.0102881 25140707 PMC4139200

[B9] Carpenter J. Connolly C. Myers C. K. (2008). Altruistic behavior in a representative dictator experiment. Exp. Econ. 11, 282–298. 10.1007/s10683-007-9193-x

[B10] Colom R. Escorial S. Shih P. C. Privado J. (2007). Fluid intelligence, memory span, and temperament difficulties predict academic performance of young adolescents. Pers. Individ. Dif. 42, 1503–1514. 10.1016/j.paid.2006.10.023

[B11] Cooper R. DeJong D. V. Forsythe R. Ross T. W. (1996). Cooperation without reputation: experimental evidence from prisoner's dilemma games. Games Econ. Behav. 12, 187–218. 10.1006/game.1996.0013

[B12] Cordero A. Corral S. (2006). DAT – 5 Tests de Aptitudes Diferenciales. Madrid: TEA Ediciones.

[B13] Dreber A. Fudenberg D. Rand D. G. (2014). Who cooperates in repeated games: the role of altruism, inequity aversion, and demographics. J. Econ. Behav. Organ. 98, 41–55. 10.1016/j.jebo.2013.12.007

[B14] Duffy S. Smith J. (2014). Cognitive load in the multi-player prisoner's dilemma game: are there brains in games? J. Behav. Exp. Econ. 51, 47–56. 10.1016/j.socec.2014.01.006

[B15] Engel C. (2011). Dictator games: a meta study. Exp. Econ. 14, 583–610. 10.1007/s10683-011-9283-7

[B16] Fischbacher U. (2007). Z-Tree: zurich toolbox for ready-made economic experiments. Exp. Econ. 10, 171–178. 10.1007/s10683-006-9159-4

[B17] Franzen A. Pointner S. (2013). The external validity of giving in the dictator game. Exp. Econ. 16, 155–169. 10.1007/s10683-012-9337-5

[B18] Greiner B. (2015). Subject pool recruitment procedures: organizing experiments with ORSEE. J. Econ. Sci. Assoc. 1, 114–125. 10.1007/s40881-015-0004-4

[B19] Harrison P. L. (1987). Research with adaptive behavior scales. J. Spec. Educ. 21, 37–68. 10.1177/002246698702100108

[B20] Hilbe C. Röhl T. Milinski M. (2014). Extortion subdues human players but is finally punished in the prisoner's dilemma. Nat. Commun. 5:3976. 10.1038/ncomms4976 24874294 PMC4050275

[B21] Jones G. (2008). Are smarter groups more cooperative? Evidence from prisoner's dilemma experiments, 1959-2003. J. Econ. Behav. Organ. 68, 489–497. 10.1016/j.jebo.2008.06.010

[B22] Jones G. (2013). Are Smarter Groups More Cooperative? Results for Corrected and Extended Datasets. Working Paper George Mason University.

[B23] Jones M. T. (2014). Strategic complexity and cooperation: an experimental study. J. Econ. Behav. Organ. 106, 352–366. 10.1016/j.jebo.2014.07.005

[B24] Kagel J. McGee P. (2014). Personality and cooperation in finitely repeated prisoner's dilemma games. Econ. Lett. 124, 274–277. 10.1016/j.econlet.2014.05.034

[B25] Keith T. Z. Fherman P. G. Harrison P. L. Pottebaum S. M. (1987). The relation between adaptive behavior and intelligence: testing alternative explanations. J. Sch. Psychol. 25, 31–43. 10.1016/0022-4405(87)90058-6

[B26] Kreps D. M. Milgrom P. Roberts J. Wilson R. (1982). Rational cooperation in the finitely repeated prisoners' dilemma. J. Econ. Theory 27, 245–252. 10.1016/0022-0531(82)90029-1

[B27] McGrew K. S. (2009). CHC theory and the human cognitive abilities project: standing on the shoulders of the giants of psychometric intelligence research. Intelligence 37, 1–10. 10.1016/j.intell.2008.08.004

[B28] Peysakhovich A. Nowak M. A. Rand D. G. (2014). Humans display a “cooperative phenotype” that is domain general and temporally stable. Nat. Commun. 5:4939 10.1038/ncomms5939 25225950

[B29] Platt L. O. Kamphaus R. W. Cole R. W. Smith C. L. (1991). Relationship between adaptive behavior and intelligence: additional evidence. Psychol. Rep. 68, 139–145. 10.2466/pr0.1991.68.1.139

[B30] Pothos E. M. Perry G. Corr P. J. Matthew M. R. Busemeyer J. R. (2011). Understanding cooperation in the Prisoner's Dilemma game. Pers. Individ. Dif. 51, 210–215. 10.1016/j.paid.2010.05.002

[B31] Proto E. Rustichini A. Sofianos A. (2015). Higher Intelligence Groups Have Higher Cooperation Rates in the Repeated Prisoner's Dilemma. CAGE Working Paper 255.

[B32] Van Lange P. A. M. De Bruin E. M. N. Otten W. Joireman J. A. (1997). Development of prosocial, individualistic, and competitive orientations: theory and preliminary evidence. J. Pers. Soc. Psychol. 73, 733–746. 10.1037/0022-3514.73.4.733 9325591

[B33] Yamagishi T. Li Y. Takagishi H. Matsumoto Y. Kiyonari T. (2014). In search of Homo economicus. Psychol. Sci. 25, 1699–1711. 10.1177/0956797614538065 25037961

