# Supplementary material for: Individual Characteristics vs. Experience: An Experimental Study on Cooperation in Prisoner's Dilemma
Source: Front Psychol. 2017 Apr 20;8:596. doi: 10.3389/fpsyg.2017.00596 (PMC5397528; doi:10.3389/fpsyg.2017.00596)
Supplement: Supplementary file 1 [file DataSheet1.docx]

Supplementary Material

Individual characteristics vs. experience: an experimental study on cooperation in Prisoner’s Dilemma

Iván Barreda-Tarrazona ^a,b,*^, Ainhoa Jaramillo-Gutiérrez ^a^,

Marina Pavan ^a^ and Gerardo Sabater-Grande ^a^

^a^ LEE & Economics Department, Universitat Jaume I, Castellón, Spain.

^b^ CERME, Management and Economics Departments, Università Ca’Foscari, Venezia, Italy.

*** Correspondence:**ivan.barreda@eco.uji.es

# Experiment Instructions (Translated from Spanish)

SESSION 1 - GENERAL INSTRUCTIONS

• The objective of this experiment is studying how individuals behave in certain economic contexts. The experiment consists of two sessions: the first today, and a second one next week, to which you will be called during the weekend.

• In today session you will have to carry out two tasks. First, you will have to take an “abstract reasoning” test, consisting of 40 questions to be answered in 20 minutes. For each correct answer, you will gain 0.25€. You will not lose any amount if your answer is incorrect. It is very important that you regularly save your answers in the computer. Especially, remember to save them before time is up, because later you will not be able to do it anymore.

• The second task is of an economic nature. The amount of money you gain in this task will be added to the one you obtained in the test to determine your total gains in this session.

• You will be paid this session’s gains confidentially next week, at the end of the second session, together with the gains you obtain in that session.

• It is very important that you ask any questions you might have by raising your hand. Apart from these questions, any type of communication with other participants is strictly prohibited and subject to exclusion from the experiment.

SESSION 1 - ECONOMIC TASK INSTRUCTIONS

In this game each participant will play two roles: the “sender” and the “recipient”. The game consists in splitting 10€ and the sender will have an active role while the recipient a passive one. In the sender role you will have to decide how much of the 10€ you want to transfer to the recipient (rounding up to one decimal at most). You will keep the rest. In the recipient role you will not have to make any decision. You will simply be informed of the amount an anonymous sender chose to transfer you. You will play both roles. Then you will be randomly paid for only one of the two roles.

Take into account that the receiver of the money you decide to transfer when you play as a sender will never be the sender when you play as a recipient. That is, you will play each role with a different partner.

SESSION 2 - GENERAL INSTRUCTIONS

• In today’s session you will carry out four economic tasks. You will be paid for only one of them, randomly chosen, once you have completed them all. The corresponding gains will be added to what you obtained in the previous session last week and you will be paid in cash, confidentially, at the end of this session.

• A voluntary participant will do the random selection to decide which task will be paid. First, the volunteer will toss a coin: if it is head, you will be paid for task 1, if it is tail, for tasks 2, 3 or 4.

• If it is head, another volunteer will draw a number from a vase containing the 10 numbers that correspond to the 10 periods of Task 1. You will be paid the gains you obtained in the period corresponding to the drawn number.

• If it is tail, another volunteer will draw a number from a vase containing the 3 numbers corresponding to Tasks 2, 3 and 4. You will be paid the accumulated gains you obtained in the task that corresponds to that number.

• It is very important that you ask any questions you might have by raising your hand. Apart from these questions, any type of communication with other participants is strictly prohibited and subject to exclusion from the experiment.

SESSION 2 - INSTRUCTIONS TASK 1

In this task you are going to play a game 10 times, but each time with a different partner, randomly selected among the participants in this session. Nobody knows the identity of the person he/she is playing with.

In this game you have to choose either A or B knowing that:

• If both players choose A, you gain 20€ and the other person gains 20€.

• If you choose A, and the other chooses B, you gain 0€ and the other gains 28€.

• If you choose B and the other chooses A, you gain 28€ and the other gains 0€.

• If both players choose B, you gain 10€ and the other gains 10€.

In the moment you choose between A and B you do not know what your partner’s decision is, and vice versa. That is, you both make your decision simultaneously, without knowing the other’s decision. After you both decided, you will be able to see what your choices and those of your partner were and the gains you obtained in that period.

You will then be randomly re-assigned a partner for the following round.

If this task is selected with the toss of the coin at the end of the experiment, your gains will correspond to the ones obtained in one of the 10 repeated games, randomly selected (with the procedure of the vase containing 10 numbers, from which one is to be drawn).

Before you start each round of this task, you will be asked to make two predictions on the behavior of the participants in this session. If your predictions turn out to be correct, you can gain up to 2 additional euros.

The first prediction is on how you think your partner will behave in each period. If you get it right, you gain 1€, zero otherwise.

The second prediction is on the behavior of all participants in the session. You will have to guess what percentage of them will choose option A. Your gains will be 100 cents of a euro (1€) minus the difference in absolute value between your predicted percentage and the real one.

The gains for these predictions will be added to the ones obtained in the economic game to determine your total gains in each period of this task.

You will not know if your predictions were correct while the session is running, only at the end of the session, when the gains relative to each task will be calculated and showed to you. This is so for each task.

SESSION 2 - INSTRUCTIONS TASKS 2, 3 AND 4

In each one of these tasks you will play a game for several rounds with a same partner. A new partner will be randomly selected in each task. Neither of you will know the identity of the other. Specifically, your partner will be randomly selected in each task and you will play with this partner during the whole duration of each task, which is of 10 rounds.

In this game you will have to decide between A and B knowing that:

• If you both choose A, you gain 2€ and the other gains 2€

• If you choose A and the other chooses B, you gain 0€ and the other gains 2.80€

• If you choose B and the other chooses A, you gain 2.80€ and the other gains 0€

• If you both choose B, you gain 1€ and the other gains 1€

In the moment you choose between A and B you do not know what your partner’s decision is, and vice versa. This information, detailed for all periods you have played with that same partner, will be given to you before you choose between A and B in the following period of that task. The game will be repeated 10 times, with the same partner.

If this task is selected (with the procedure described in the general instructions), your gains will be the sum of what you obtained in the 10 periods played with the same partner in this task.

This repeated game will be played 3 times, one in each task, that is, after playing 10 periods with the same partner in Task 2, you will play with a randomly selected partner for another 10 periods. After these 10 rounds, you will play for another last 10 times with a newly selected partner in Task 4.

Before each period of these tasks, you will be asked to make two predictions on the behavior of the participants in that period. If your predictions are correct, you can gain up to 2 additional euros accumulated at the end of the task.

The first prediction is on how you think your partner will behave in each period. You gain 0.1€ for each period in which your prediction is correct, zero for each period in which your prediction is not correct.

The second prediction is on the behavior of all participants in each period of the session. You will have to guess what percentage of them will choose option A. Each period, your gains will be 10 cents of a euro (0.1€) minus one tenth of the difference in absolute value between your predicted percentage and the real one.

The gains for these predictions in a task will be added to the ones obtained in the economic game of the same task to determine your total gains in that task.

# Supplementary Tables and Figures

This section contains detailed results from the inferential analysis. We perform a Bonferroni correction to take into account the problem of false positives in multiple comparisons. In particular, this correction consists in multiplying the p-value by the number of comparisons using the same dataset in each period, resulting in a rather demanding threshold for rejection.

In Tables SM2.1 to SM2.4, we compare the individual beliefs of “High Altruism and Low Reasoning Ability” (HALR) subjects with each of the other Treatments’ individual beliefs in all periods of each task, using proportion tests.

**Table SM2.1.** Statistical comparisons of the individual expectation of partner’s cooperation in the current period, in task 1, using proportion tests.

Dark green: significant at 5%. Light green: significant at 10%. No color: no significant differences (=) or not possible to conduct the test (-).

| Period | HALR vs LALR | HALR vs LAHR | HALR vs HAHR |
| --- | --- | --- | --- |
| 1 | z = 2.62  p = 0.027 | z = 3.83  p < 0.001 | z = 2.61  p = 0.027 |
| 2 | z = 2.42  p = 0.048 | z = 3.20  p = 0.003 | z = 2.00  p = 0.135 |
| 3 | z = 2.27  p = 0.069 | z = 3.72  p < 0.001 | z =1.93  p = 0.162 |
| 4 | z = 2.56  p = 0.030 | z = 3.49  p < 0.001 | z = 1.66  p = 0.288 |
| 5 | z = 2.23  p = 0.069 | z = 2.36  p = 0.054 | z = 1.36  p = 0.522 |
| 6 | z = 1.69  p = 0.270 | z = 2.41  p = 0.054 | z = 0.82  p = 1.000 |
| 7 | z = 2.76  p = 0.018 | z = 2.89  p = 0.012 | z = 1.86  p = 0.189 |
| 8 | z = 1.43  p = 0.456 | z = 1.50  p = 0.402 | z = 1.25  p = 0.633 |
| 9 | z = 1.76  p = 0.234 | z = 1.84  p = 0.195 | z = 0.76  p = 1.000 |
| 10 | z = 2.05  p = 0.120 | z = 2.14  p = 0.096 | z = 1.09  p = 0.831 |

**Table SM2.2.** Statistical comparisons of the individual expectation of partner’s cooperation in the current period, in task 2, using proportion tests.

Dark green: significant at 5%. Light green: significant at 10%. No color: no significant differences (=) or not possible to conduct the test (-).

| Period | HALR vs LALR | HALR vs LAHR | HALR vs HAHR |
| --- | --- | --- | --- |
| 1 | z = 2.19  p = 0.084 | z = 3.64  p < 0.001 | z = 1.41  p = 0.477 |
| 2 | z = 0.22  p = 1.000 | z = 1.21  p = 0.681 | z = 0.26  p = 1.000 |
| 3 | z = 0.66  p = 1.000 | z = 0.55  p = 1.000 | z = -0.28  p = 1.000 |
| 4 | z = 0.44  p = 1.000 | z = 0.99  p = 0.963 | z = 0.19  p = 1.000 |
| 5 | z =1.32  p = 0.558 | z =0.33  p = 1.000 | z = -0.70  p = 1.000 |
| 6 | z = 0.44  p = 1.000 | z = 0.10  p = 1.000 | z = 0.05  p = 1.000 |
| 7 | z = 0.68  p = 1.000 | z = 0.56  p = 1.000 | z = -0.65  p = 1.000 |
| 8 | z = 0.46  p = 1.000 | z = 0.80  p = 1.000 | z = -0.86  p = 1.000 |
| 9 | z = 0.46  p = 1.000 | z = 0.56  p = 1.000 | z = -0.37  p = 1.000 |
| 10 | z = 1.06  p = 0.861 | z = 0.60  p = 1.000 | z = -1.48  p = 0.420 |

**Table SM2.3.** Statistical comparisons of the individual expectation of partner’s cooperation in the current period, in task 3, using proportion tests.

Dark green: significant at 5%. Light green: significant at 10%. No color: no significant differences (=) or not possible to conduct the test (-).

| Period | HALR vs LALR | HALR vs LAHR | HALR vs HAHR |
| --- | --- | --- | --- |
| 1 | z =1.09  p = 0.722 | z = 2.96  p = 0.009 | z = 1.08  p = 0.837 |
| 2 | z = 0.00  p = 1.000 | z = 0.84  p = 1.000 | z = -0.60  p = 1.000 |
| 3 | z = 0.22  p = 1.000 | z = 0.020  p = 1.000 | z = -0.60  p = 1.000 |
| 4 | z = -0.65  p = 1.000 | z = -0.04  p = 1.000 | z = -0.41  p = 1.000 |
| 5 | z = 0.44  p = 1.000 | z = -0.65  p = 1.000 | z = -0.94  p = 1.000 |
| 6 | z = -0.65  p = 1.000 | z = -0.45  p = 1.000 | z = -0.67  p = 1.000 |
| 7 | z = -0.66  p = 1.000 | z = -0.87  p = 1.000 | z = -0.88  p = 1.000 |
| 8 | z = 0.44  p = 1.000 | z = -0.65  p = 1.000 | z = -0.41  p = 1.000 |
| 9 | z = -0.44  p = 1.000 | z =-0.67  p = 1.000 | z = -0.34  p = 1.000 |
| 10 | z = -0.25  p = 1.000 | z =0.48  p = 1.000 | z = -0.42  p = 1.000 |

**Table SM2.4.** Statistical comparisons of the individual expectation of partner’s cooperation in the current period, in task 4, using proportion tests.

Dark green: significant at 5%. Light green: significant at 10%. No color: no significant differences (=) or not possible to conduct the test (-).

| Period | HALR vs LALR | HALR vs LAHR | HALR vs HAHR |
| --- | --- | --- | --- |
| 1 | z = 0.68  p = 1.000 | z = 2.58  p = 0.030 | z = 1.45  p = 0.444 |
| 2 | z = -1.98  p = 0.144 | z =-1.26  p = 0.621 | z = -1.47  p = 0.423 |
| 3 | z = -2.41  p = 0.048 | z = -1.91  p = 0.168 | z = -2.69  p = 0.021 |
| 4 | z = -1.32  p = 0.558 | z = -1.03  p = 0.915 | z = -1.07  p = 0.852 |
| 5 | z = -1.75  p = 0.237 | z = -1.47  p = 0.426 | z = -1.47  p = 0.423 |
| 6 | z = -1.55  p = 0.363 | z = -1.03  p = 0.915 | z = -1.07  p = 0.852 |
| 7 | z = -1.75  p = 0.243 | z = -1.69  p = 0.273 | z = -1.41  p = 0.477 |
| 8 | z = -0.88  p = 1.000 | z = -0.80  p = 1.000 | z = -0.33  p = 1.000 |
| 9 | z = -0.87  p = 1.000 | z = -0.84  p = 1.000 | z = -0.47  p = 1.000 |
| 10 | z = -0.26  p = 1.000 | z = 0.48  p = 1.000 | z = 0.28  p = 1.000 |

Figure SM2.1 presents the mean percentage of individuals expected to cooperate in each period (the “social belief”, that is, the answer to the second question reported in Section 2.3), by task, period and treatment. In this case again, it is HALR subjects who exhibit the most optimistic view of cooperation in their session, consistently over tasks and time. When comparing the HALR treatment with the rest using a Mann-Whitney test, we find that it is significant in all periods only for task 1. Regarding HALR subjects in the RPD games, we find significant differences only when we compare them with LAHR players: for all periods in task 2 and for the first periods in tasks 3 and 4 (the results of these tests are available upon request).

**Figure SM2.1** Mean percentage of individuals expected to choose cooperation in the current period by task, period and treatment.

The following four Tables present the statistical comparisons of percentages of correct predictions between treatments in the four tasks, by period.

**Table SM2.5.** Statistical comparisons of percentages of subjects who correctly predict their partner's decision in the one-shot game per treatment, using proportion tests.

Dark green: significant at 5%. Light green: significant at 10%. No color: no significant differences (=) or not possible to conduct the test (-).

| Period | LR vs HR  (1) | LALR vs LAHR  (2) | HALR vs HAHR  (3) | LA vs HA  (4) | LALR vs HALR  (5) | LAHR vs HAHR  (6) |
| --- | --- | --- | --- | --- | --- | --- |
| 1 | z = -3.15  p < 0.001 | z = -2.55  p = 0.030 | z = -1.74  p = 0.240 | z = 1.53  p = 0.744 | z = 0.65  p = 1.000 | z = 1.23  p = 1.000 |
| 2 | z = -2.59  p = 0.024 | z = -1.30  p = 0.570 | z = -2.08  p = 0.108 | z = 2.45  p = 0.084 | z = 2.06  p = 0.234 | z = 1.02  p = 1.000 |
| 3 | z = -3.59  p < 0.001 | z = -1.68  p = 0.276 | z = -3.06  p = 0.006 | z = 3.01  p = 0.012 | z = 2.73  p = 0.018 | z = 0.94  p = 1.000 |
| 4 | z = -2.27  p = 0.066 | z = -0.51  p = 1.000 | z = -2.12  p = 0.096 | z = 3.42  p < 0.001 | z = 3.07  p = 0.012 | z = 1.30  p = 1.000 |
| 5 | z = -1.74  p = 0.240 | z = -0.668  p = 1.000 | z = -1.39  p = 0.486 | z = 2.80  p = 0.030 | z = 2.26  p = 0.138 | z = 1.41  p = 0.936 |
| 6 | z = -2.26  p = 0.066 | z = -1.49  p = 0.402 | z = -1.59  p = 0.330 | z = 2.72  p = 0.018 | z = 2.02  p = 0.258 | z = 1.71  p = 0.510 |
| 7 | z = -1.88  p = 0.174 | - | z = -1.61  p = 0.318 | z = 4.09  p < 0.001 | z = 3.36  p < 0.001 | z = 2.11  p = 0.204 |
| 8 | z = -0.93  p = 1.000 | - | z = -0.75  p = 1.000 | z = 2.20  p = 0.182 | z = 1.76  p = 0.462 | z = 1.20  p = 1.000 |
| 9 | z = -1.57  p = 0.392 | - | z = -1.37  p = 0.510 | z = 2.72  p = 0.018 | z = 2.30  p = 0.126 | z = 1.20  p = 1.000 |
| 10 | z = -2.06  p = 0.114 | - | z = -1.85  p = 0.186 | z = 3.16  p < 0.001 | z = 2.76  p = 0.030 | z = 1.20  p = 1.000 |

**Table SM2.6.** Statistical comparisons of percentages of subjects who correctly predict their partner's decision in task 2 per treatment, using proportion tests.

Dark green: significant at 5%. Light green: significant at 10%. No color: no significant differences (=) or not possible to conduct the test (-).

| Period | LR vs HR  (1) | LALR vs LAHR  (2) | HALR vs HAHR  (3) | LA vs HA  (4) | LALR vs HALR  (5) | LAHR vs HAHR  (6) |
| --- | --- | --- | --- | --- | --- | --- |
| 1 | z = -2.54  p = 0.030 | z = -1.67  p = 0.276 | z = -1.87  p = 0.180 | z = 0.57  p = 1.000 | z = 0.44  p = 1.000 | z = 0.04  p = 1.000 |
| 2 | z = -0.45  p = 1.000 | z = -0.73  p = 1.000 | z = 0.11  p = 1.000 | z = -0.09  p = 1.000 | z = -0.49  p = 1.000 | z = 0.33  p = 1.000 |
| 3 | z = -0.80  p = 1.000 | z = -1.61  p = 0.318 | z = 0.56  p = 1.000 | z = 0.30  p = 1.000 | z = -0.814  p = 1.000 | z = 1.32  p = 1.000 |
| 4 | z = -1.63  p = 0.306 | z = -1.49  p = 0.402 | z = -0.82  p = 1.000 | z = -0.39  p = 1.000 | z = -0.62  p = 1.000 | z = -0.04  p = 1.000 |
| 5 | z = -0.83  p = 1.000 | z = -1.61  p = 0.312 | z = 0.46  p = 1.000 | z = 0.89  p = 1.000 | z = -0.32  p = 1.000 | z = 1.71  p = 0.516 |
| 6 | z = -3.04  p = 0.006 | z = -2.88  p = 0.012 | z = -1.37  p = 0.510 | z = 0.03  p = 1.000 | z = -0.62  p = 1.000 | z = 1.20  p = 1.000 |
| 7 | z = -1.57  p = 0.342 | z = -1.05  p = 0.876 | z = -1.08  p = 0.828 | z = 1.88  p = 0.354 | z = 1.38  p = 1.000 | z = 1.20  p = 1.000 |
| 8 | z = -1.83  p = 0.198 | z = -1.49  p = 0.402 | z = -1.08  p = 0.828 | z = 1.39  p = 0.972 | z = 0.84  p = 1.000 | z = 1.20  p = 1.000 |
| 9 | z = -0.61  p = 1.000 | z = -0.66  p = 1.000 | z = -0.15  p = 1.000 | z = 0.97  p = 1.000 | z = 0.46  p = 1.000 | z = 0.92  p = 1.000 |
| 10 | z = 0.43  p = 1.000 | z = 0.31  p = 1.000 | z = 0.299  p = 1.000 | z = -0.02  p = 1.000 | z = 0.00  p = 1.000 | z = 0.01  p = 1.000 |

**Table SM2.7.** Statistical comparisons of percentages of subjects who correctly predict their partner's decision in task 3 per treatment, using proportion tests.

Dark green: significant at 5%. Light green: significant at 10%. No color: no significant differences (=) or not possible to conduct the test (-).

| Period | LR vs HR  (1) | LALR vs LAHR  (2) | HALR vs HAHR  (3) | LA vs HA  (4) | LALR vs HALR  (5) | LAHR vs HAHR  (6) |
| --- | --- | --- | --- | --- | --- | --- |
| 1 | z = 0.94  p = 1.000 | z = 0.44  p = 1.000 | z = 1.00  p = 1.000 | z = 0.63  p = 1.000 | z = 0.21  p = 1.000 | z = 0.81  p = 1.000 |
| 2 | z = 1.87  p = 1.000 | z = 1.89  p = 1.000 | z = 1.13  p = 1.000 | z = 2.26  p = 0.138 | z = 2.12  p = 0.198 | z = 1.41  p = 0.942 |
| 3 | z = 0.63  p = 1.000 | z = 0.55  p = 1.000 | z = 0.07  p = 1.000 | z = -1.58  p = 0.678 | z = -0.90  p = 1.000 | z = -1.25  p = 1.000 |
| 4 | z = -0.15  p = 1.000 | z = -0.87  p = 1.000 | z = 0.78  p = 1.000 | z = -0.54  p = 1.000 | z = -1.18  p = 1.000 | z = 0.465  p = 1.000 |
| 5 | z = -2.06  p = 0.114 | z = -2.14  p = 0.096 | z = -0.75  p = 1.000 | z = 0.25  p = 1.000 | z = -0.39  p = 1.000 | z = 1.20  p = 1.000 |
| 6 | z = -2.06  p = 0.114 | z = -2.36  p = 0.054 | z = -0.35  p = 1.000 | z = -1.26  p = 1.000 | z = -1.76  p = 0.462 | z = 0.26  p = 1.000 |
| 7 | z = -2.06  p = 0.114 | z = -2.14  p = 0.096 | z = -0.75  p = 1.000 | z = 0.25  p = 1.000 | z = 0.00  p = 1.000 | z = 1.20  p = 1.000 |
| 8 | z = -0.51  p = 1.000 | z = -0.06  p = 1.000 | z = -0.87  p = 1.000 | z = -0.43  p = 1.000 | z = 0.00  p = 1.000 | z = -0.83  p = 1.000 |
| 9 | z = 0.12  p = 1.000 | z = -0.09  p = 1.000 | z = 0.34  p = 1.000 | z = 0.93  p = 1.000 | z = 0.461  p = 1.000 | z = 0.89  p = 1.000 |
| 10 | z = -0.74  p = 1.000 | z = -0.747  p = 1.000 | z = -0.34  p = 1.000 | z = -0.35 p = 1.000 | z = -0.472  p = 1.000 | z = -0.10  p = 1.000 |

**Table SM2.8.** Statistical comparisons of percentages of subjects who correctly predict their partner's decision in task 4 per treatment, using proportion tests.

Dark green: significant at 5%. Light green: significant at 10%. No color: no significant differences (=) or not possible to conduct the test (-).

| Period | LR vs HR  (1) | LALR vs LAHR  (2) | HALR vs HAHR  (3) | LA vs HA  (4) | LALR vs HALR  (5) | LAHR vs HAHR  (6) |
| --- | --- | --- | --- | --- | --- | --- |
| 1 | z = -0.280  p = 1.000 | z = -0.30  p = 1.000 | z = -0.33  p = 1.000 | z = -1.81  p = 0.414 | z = -1.31  p = 1.000 | z = -1.30  p = 1.000 |
| 2 | z = 0.44  p = 1.000 | z = 0.55  p = 1.000 | z = 0.11  p = 1.000 | z = 0.41  p = 1.000 | z = 0.53  p = 1.000 | z = 0.11  p = 1.000 |
| 3 | z = -0.48  p = 1.000 | z = -1.77  p = 0.228 | z = 1.15  p = 1.000 | z = 0.67  p = 1.000 | z = -0.90  p = 1.000 | z = 1.99  p = 0.276 |
| 4 | z = -0.60  p = 1.000 | z = -1.84  p = 0.192 | z = 0.56  p = 1.000 | z = 3.20  p = 0.006 | z = 1.34  p = 1.000 | z = 3.32  p < 0.001 |
| 5 | z = -1.42  p = 0.462 | z = -1.48  p = 0.408 | z = -0.49  p = 1.000 | z = 1.95  p = 0.306 | z = 0.970  p = 1.000 | z = 1.83  p = 0.402 |
| 6 | z = -1.05  p = 0.144 | z = -1.49  p = 0.402 | z = -0.15  p = 1.000 | z = 1.39  p = 0.972 | z = 0.46  p = 1.000 | z = 1.71  p = 0.510 |
| 7 | z = -1.05  p = 0.864 | z = -1.49  p = 0.402 | z = -0.15  p = 1.000 | z = 1.39  p = 0.972 | z = 0.46  p = 1.000 | z = 1.71  p = 0.510 |
| 8 | z = -1.83  p = 0.198 | z = -1.11  p = 0.786 | z = -1.54  p = 0.366 | z = -0.15  p = 1.000 | z = 0.00  p = 1.000 | z = -0.83  p = 1.000 |
| 9 | z = 0.63  p = 1.000 | z = 0.80  p = 1.000 | z = 0.23  p = 1.000 | z = 0.90  p = 1.000 | z = 0.97  p = 1.000 | z = 0.411  p = 1.000 |
| 10 | z = -0.55  p = 1.000 | z = -0.74  p = 1.000 | z = -0.12  p = 1.000 | z = -1.03 p = 1.000 | z = -1.06  p = 1.000 | z = -0.44  p = 1.000 |

In Table SM2.9 we compare cooperation rates in period 1 between tasks within each treatment, using proportion tests.

**Table SM2.9.** Proportion tests comparing the percentage of cooperation in period 1 between tasks, by treatment.

Dark green: significant at 5%. Light green: significant at 10%. No color: no significant differences (=) or not possible to conduct the test (-).

| Tasks | LALR | LAHR | HALR | HAHR |
| --- | --- | --- | --- | --- |
| Task 1- Task 2 | z = -2.49  p = 0.052 | z = -4.04  p < 0.001 | z = -1.75  p = 0.324 | z = -2.70  p = 0.028 |
| Task 1 –Task 3 | z = -3.32  p = 0.004 | z = -4.75  p < 0.001 | z = -1.75  p = 0.324 | z = -2.93  p = 0.012 |
| Task 1 –Task 4 | z = -4.58  p < 0.001 | z = -5.61  p < 0.001 | z = -1.96  p = 0.196 | z = -3.84  p < 0.001 |
| Task 2 –Task 3 | z = -0.88  p = 1.000 | z = -0.84  p = 1.000 | z = 0.00  p = 1.000 | z = -0.25  p = 1.000 |
| Task 3 –Task 4 | z = -1.39  p = 0.660 | z = -1.04  p = 1.000 | z = -0.22  p = 1.000 | z = -1.01  p = 1.000 |

In Tables SM2.10 and SM2.11 we present the percentages used to elaborate Figure 6 in the main text.

**Table SM2.10.** Percentages of individual cooperation in the one-shot PD game per treatment.

| Period | LALR | LAHR | HALR | HAHR |
| --- | --- | --- | --- | --- |
| 1 | 23.81 | 4.35 | 40.48 | 15.63 |
| 2 | 7.14 | 2.17 | 23.81 | 3.13 |
| 3 | 11.90 | 4.35 | 28.57 | 12.50 |
| 4 | 2.38 | 4.35 | 19.05 | 3.13 |
| 5 | 2.38 | 0.00 | 4.76 | 3.13 |
| 6 | 2.38 | 0.00 | 11.90 | 0.00 |
| 7 | 0.00 | 0.00 | 7.14 | 6.25 |
| 8 | 0.00 | 0.00 | 2.38 | 3.13 |
| 9 | 0.00 | 0.00 | 4.76 | 0.00 |
| 10 | 0.00 | 0.00 | 7.14 | 0.00 |

**Table SM2.11.** Percentages of individual cooperation in the RPD game per treatment and task.

|  | LALR | | | LAHR | | | HALR | | | HAHR | | |
| --- | --- | --- | --- | --- | --- | --- | --- | --- | --- | --- | --- | --- |
| Period | Task 2 | Task 3 | Task 4 | Task 2 | Task 3 | Task 4 | Task 2 | Task 3 | Task 4 | Task 2 | Task 3 | Task 4 |
| 1 | 50.00 | 59.52 | 73.81 | 39.13 | 47.83 | 58.70 | 59.52 | 59.52 | 61.90 | 46.88 | 50.00 | 62.50 |
| 2 | 52.38 | 54.76 | 71.43 | 36.96 | 50.00 | 56.52 | 40.48 | 54.76 | 50.00 | 50.00 | 59.38 | 62.50 |
| 3 | 47.62 | 59.52 | 71.43 | 36.96 | 50.00 | 63.04 | 38.10 | 50.00 | 47.62 | 46.88 | 59.38 | 62.50 |
| 4 | 45.24 | 54.76 | 66.67 | 34.78 | 52.17 | 60.87 | 40.48 | 45.24 | 52.38 | 40.63 | 53.13 | 65.63 |
| 5 | 40.48 | 54.76 | 64.29 | 39.13 | 52.17 | 63.04 | 38.10 | 47.62 | 57.14 | 46.88 | 53.13 | 62.50 |
| 6 | 40.48 | 54.76 | 61.90 | 39.13 | 52.17 | 60.87 | 30.95 | 45.24 | 52.38 | 40.63 | 50.00 | 56.25 |
| 7 | 38.10 | 50.00 | 61.90 | 39.13 | 52.17 | 60.87 | 28.57 | 50.00 | 50.00 | 37.50 | 50.00 | 59.38 |
| 8 | 35.71 | 47.62 | 59.52 | 39.13 | 50.00 | 58.70 | 30.95 | 42.86 | 50.00 | 37.50 | 50.00 | 56.25 |
| 9 | 33.33 | 42.86 | 47.62 | 36.96 | 45.65 | 45.65 | 26.19 | 40.48 | 40.48 | 37.50 | 43.75 | 40.63 |
| 10 | 16.67 | 16.67 | 7.14 | 17.39 | 10.87 | 2.17 | 16.67 | 4.76 | 19.05 | 21.88 | 9.38 | 3.13 |

Table SM2.12 presents proportion tests statistics comparing individual cooperation rates between treatments in the one-shot PD (task 1).

**Table SM2.12.** Statistical comparisons of percentages of individual cooperation in the one-shot PD game using proportion tests.

Dark green: significant at 5%. Light green: significant at 10%. No color: no significant differences (=) or not possible to conduct the test (-).

| Period | LR vs HR  (1) | LALR vs LAHR  (2) | HALR vs HAHR  (3) | LA vs HA  (4) | LALR vs HALR  (5) | LAHR vs HAHR  (6) |
| --- | --- | --- | --- | --- | --- | --- |
| 1 | z = 3.62  p < 0.001 | z = 2.66  p = 0.048 | z = 2.32  p = 0.120 | z = -2.51  p = 0.036 | z = -1.64  p = 0.306 | z = -1.71  p = 0.258 |
| 2 | z = 2.83  p = 0.030 | z = 1.12  p = 1.000 | z = 2.48  p = 0.078 | z = -2.26  p = 0.072 | z = -2.11  p = 0.102 | z = -0.26  p = 1.000 |
| 3 | z = 2.29  p = 0.132 | z = 1.31  p = 1.000 | z = 1.66  p = 0.576 | z = -2.48  p = 0.042 | z = -1.90  p = 0.174 | z = -1.33  p = 0.552 |
| 4 | z = 1.67  p = 0.570 | z = -0.51  p = 1.000 | z = 2.08  p = 0.228 | z = -2.12  p = 0.102 | z = -2.47  p = 0.042 | z = 0.28  p = 1.000 |
| 5 | z = 0.94  p = 1.000 | z = 1.05  p = 1.000 | z = 0.35  p = 1.000 | z = -1.19 p = 0.233 | z = -0.59  p = 1.000 | - |
| 6 | z = 2.41  p = 0.096 | z = 1.05  p = 1.000 | z = 2.02  p = 0.258 | z = -1.89  p = 0.180 | z = -1.69  p = 0.270 | - |
| 7 | z = 0.37  p = 1.000 | - | z = 0.15  p = 1.000 | z = -2.48  p = 0.042 | z = -1.76  p = 0.234 | z = -1.72  p = 0.258 |
| 8 | z = -0.05  p = 1.000 | - | z = -0.20  p = 1.000 | z = -1.55  p = 0.360 | z = -1.01  p = 1.000 | z = -1.21  p = 1.000 |
| 9 | z = 1.37  p = 1.000 | - | z = 1.25  p = 1.000 | z = -1.55  p = 0.360 | z = -1.43  p = 0.456 | - |
| 10 | z = 1.68  p = 0.552 | - | z = 1.54  p = 0.738 | z = -1.91  p = 0.168 | z = -1.76  p = 0.234 | - |

In Table SM2.13, we test for collinearity in the logistic models presented in Table 6 in the main text.

**Table SM2.13.** Variance Inflation Factor for the regressors in the models of Table 6.

R-squared in parentheses.

| Individual Cooperation | Task 1 | | Task 2 | | Task 3 | | Task 4 | |
| --- | --- | --- | --- | --- | --- | --- | --- | --- |
|  | VIF | R^2^ | VIF | R^2^ | VIF | R^2^ | VIF | R^2^ |
| Reasoning ability | 1.07 | (0.06) | 1.03 | (0.03) | 1.03 | (0.03) | 1.03 | (0.03) |
| Altruism | 1.06 | (0.06) | 1.04 | (0.04) | 1.05 | (0.04) | 1.05 | (0.05) |
| Social belief | 1.13 | (0.12) |  |  |  |  |  |  |
| Individual belief |  |  | 1.01 | (0.01) | 1.02 | (0.02) | 1.03 | (0.02) |
| Female | 1.03 | (0.03) | 1.03 | (0.03) | 1.03 | (0.03) | 1.03 | (0.03) |
| Period | 1.05 | (0.05) | 1.00 | (0.00) | 1.01 | (0.01) | 1.01 | (0.01) |
| N | 1620 |  | 1620 |  | 1620 |  | 1620 |  |
| Mean VIF | 1.07 |  | 1.02 |  | 1.03 |  | 1.03 |  |

Table SM2.14 presents Wilcoxon tests comparing paired cooperation in each pair of consecutive periods in the repeated Prisoner’s Dilemma games.

**Table SM2.14.** Statistical comparisons of paired cooperation between consecutive periods in the repeated game using Wilcoxon tests.

Dark green: significant at 5%. Light green: significant at 10%. No color: no significant differences (=) or not possible to conduct the test (-).

| Task | Task 2 | | Task 3 | | Task 4 | |
| --- | --- | --- | --- | --- | --- | --- |
| Period  Comparison | HR | LR | HR | LR | HR | LR |
| 2 vs. 1 | z = 2.00  p = 0.045 | z = 1.00  p = 0.317 | z = 2.23  p = 0.025 | z = 1.41  p = 0.157 | z = 1.89  p = 0.058 | z = 1.73  p = 0.083 |
| 3 vs. 2 | z = 1.00  p = 0.317 | z = 0.00  p = 1.000 | z = 1.89  p = 0.058 | z = 0.57  p = 0.563 | z = 2.00  p = 0.045 | z = 1.00  p = 0.317 |
| 4 vs. 3 | - | z = 1.00  p = 0.317 | z = 1.00  p = 0.317 | z = 1.00  p = 0.317 | z = 1.00  p = 0.317 | z = 1.00  p = 0.317 |
| 5 vs. 4 | z = 1.41  p = 0.157 | z = -0.57  p = 0.563 | - | z = -0.57  p = 0.563 | - | z = 1.00  p = 0.317 |
| 6 vs. 5 | - | z = -0.57  p = 0.563 | - | - | - | z = -1.00  p = 0.317 |
| 7 vs. 6 | - | - | - | - | - | - |
| 8 vs. 7 | - | z = 0.00  p = 1.000 | z = 1.00  p = 0.317 | - | z = -1.00  p = 0.317 | z = -1.00  p = 0.317 |
| 9 vs. 8 | z = -1.41  p = 0.157 | z = -1.00  p = 0.317 | z = -1.73  p = 0.083 | z = -2.00  p = 0.045 | z = -3.16  p = 0.001 | z = -2.64  p = 0.008 |
| 10 vs. 9 | z = -3.31  p < 0.001 | z = -3.00  p = 0.002 | z = -3.87  p < 0.001 | z = -3.74  p < 0.001 | z = -3.46  p < 0.001 | z = -3.60  p < 0.001 |
